# Brain mediators of biased social learning of self-perception in social anxiety disorder

**DOI:** 10.1038/s41398-023-02587-z

**Published:** 2023-09-02

**Authors:** Leonie Koban, Jessica R. Andrews-Hanna, Lindsay Ives, Tor D. Wager, Joanna J. Arch

**Affiliations:** 1 Lyon Neuroscience Research Center (CRNL), CNRS, INSERM, Université Claude Bernard Lyon 1, Bron, France; 2 Department of Psychology, University of Arizona, Tucson, AZ USA; 3 Department of Psychology and Neuroscience, University of Colorado, Boulder, CO USA; 4 Department of Cognitive and Brain Sciences, Dartmouth College, Hanover, NH USA

**Keywords:** Psychiatric disorders, Learning and memory

## Abstract

Social anxiety disorder (SAD) is characterized by an excessive fear of social evaluation and a persistently negative view of the self. Here we test the hypothesis that negative biases in brain responses and in social learning of self-related information contribute to the negative self-image and low self-esteem characteristic of SAD. Adult participants diagnosed with social anxiety (*N* = 21) and matched controls (*N* = 23) rated their performance and received social feedback following a stressful public speaking task. We investigated how positive versus negative social feedback altered self-evaluation and state self-esteem and used functional Magnetic Resonance Imaging (fMRI) to characterize brain responses to positive versus negative feedback. Compared to controls, participants with SAD updated their self-evaluation and state self-esteem significantly more based on negative compared to positive social feedback. Responses in the frontoparietal network correlated with and mirrored these behavioral effects, with greater responses to positive than negative feedback in non-anxious controls but not in participants with SAD. Responses to social feedback in the anterior insula and other areas mediated the effects of negative versus positive feedback on changes in self-evaluation. In non-anxious participants, frontoparietal brain areas may contribute to a positive social learning bias. In SAD, frontoparietal areas are less recruited overall and less attuned to positive feedback, possibly reflecting differences in attention allocation and cognitive regulation. More negatively biased brain responses and social learning could contribute to maintaining a negative self-image in SAD and other internalizing disorders, thereby offering important new targets for interventions.

## Introduction

Social anxiety disorder (SAD) is an early-onset and often chronic mental health condition with a lifetime prevalence of 12% [1]. SAD is characterized by excessive fear of social evaluation and avoidance of social interaction, which cause substantial impairment in personal, social, and professional functioning [2]. Cognitive theories propose that negative views about the self and concerns about being perceived as deficient by others are at the core of SAD [3, 4]. Indeed, individuals with SAD often rate themselves, their character, and their appearance more negatively than non-anxious individuals [5, 6]. SAD is also associated with low self-esteem and self-compassion [7, 8], low positive affect [9], and high self-criticism [10, 11].

The current study evaluates the social learning mechanisms that cause and maintain this negative view of the self in SAD. We previously developed a computational modeling approach to assess these putative mechanisms and provided initial evidence for a bias towards learning from negative (vs. positive) feedback in SAD [12]. Biased learning about self-related information and biased updating of state self-esteem in SAD could contribute to the maintenance of negative self-view and low self-esteem core to the disorder. Other behavioral and computational studies have replicated and extended these findings [13–17]. Further, a recent study suggests that negative learning bias is predictive of future symptoms of anxiety [18]. However, little is known about the brain mechanisms that underlie the biased processing of positive versus negative self-related social information in SAD. Here, we use fMRI to investigate the brain mechanisms of biased learning about the self, in adults diagnosed with SAD and in non-anxious control participants.

Previous studies point to several functional brain systems altered in SAD and other anxiety disorders that may contribute to biased learning of self-perception. An early meta-analysis of fMRI studies showed that SAD, compared to non-SAD control participants, are characterized by increased activation of limbic areas related to affect such as amygdala and anterior insula [19]. Several previous studies and theoretical frameworks have proposed that SAD, as well as other anxiety disorders, exhibit altered functionality of the dorsolateral prefrontal cortex or the frontoparietal network (FPN). An influential finding is hypoactivation of lateral prefrontal areas in anxiety [20], consistent with the possibility that highly anxious individuals are impaired in cognitive control and emotion regulation processes. Such regulatory processes can both guide attention away from anxiety-provoking information and regulate affective appraisal [21, 22]. Yet not all studies find reductions in prefrontal or frontoparietal activity, and a meta-analysis [23] suggests mixed effects in frontal areas. Thus, an alternative hypothesis is that highly anxious people may engage brain regions involved in attentional and emotional control in different and less adaptive ways. In other words, highly anxious participants may engage FPN and other regions to maintain attention on anxiety-provoking or negative stimuli, instead of optimally diverting attention *away* from them. If so, FPN activity may respond more strongly to negative information in individuals with SAD and to positive information in non-anxious controls.

The FPN is involved in cognitive control, working memory, attention, and other executive processes [24]. It also has a central role in emotion regulation [22, 25] as well as in different types of instruction and suggestion effects [26]. For example, we recently showed that frontoparietal activity mediated social influence effects on changes in pain ratings [27]. Activity of the FPN may also mediate social feedback effects on self-perception and self-esteem. A recent model of self-related processing in the brain [28] suggests that the default mode network (DMN) represents self-related content and that a valuation network (comprising insula, midcingulate cortex, and limbic areas) codes the negative and positive valence of these beliefs. The FPN is thought to subserve the context-dependent and meta-cognitive regulation of these self-related beliefs and affect [28].

**Fig. 1:**
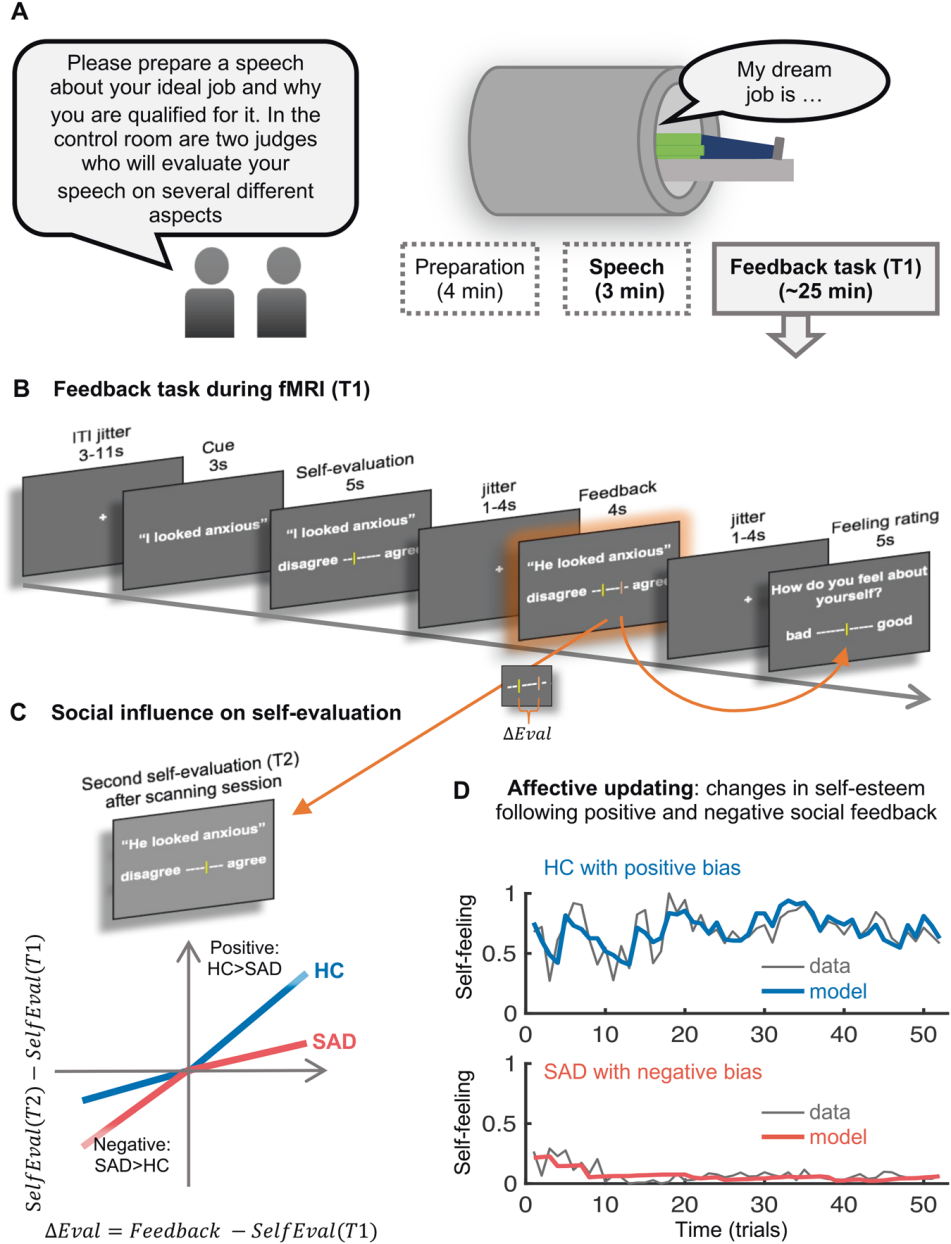
Overview of the experimental procedures, design of feedback task, and measures of interest. **A** Overview. While in the MRI scanner, participants were told to prepare a short (3 minutes) speech to be given to two judges (confederates) who would evaluate their performance and give them feedback later. Participants had 4 min for speech preparation and then gave their speech using the scanner interphone. If the participant remained silent for more than 20 seconds, one of the judges prompted them via the scanner interphone (“Please continue”). The feedback task followed the speech after a brief break. **B** Trial design of the feedback task. Each of 52 trials started with a short presentation of an evaluative cue, written in a first-person perspective (e.g., “I looked anxious”, “I was convincing”). Participants rated how much they thought this applied to their speech performance, and then received feedback from the judges on the same visual analogue scale (VAS) but written in third-person perspective (e.g., “He looked anxious”, “She was convincing”). This feedback was drawn from a distribution centered on the participant’s self-evaluation, resulting in a feedback mismatch (Δ*Eval*, difference between social feedback and initial self-evaluation) that was either more positive or more negative than the self-evaluation**. C** Hypothesized group differences in positive versus negative adjustments. We predicted that HC (in blue) would learn more (reflected in steeper slopes/larger beta weights) from positive compared to negative feedback, while SAD (in red) would learn more based on negative compared to positive feedback. **D** Measuring state self-esteem (‘How do you feel about yourself?’) at the end of each trial allowed us to fit an adapted Rescorla-Wagner learning model that described how positive and negative social feedback impacted self-esteem over time (‘affective updating’). The plots show the time course of state self-esteem (rating data and modeled data) from two example subjects, with high positive and high negative affective updating biases, respectively.

**Fig. 2:**
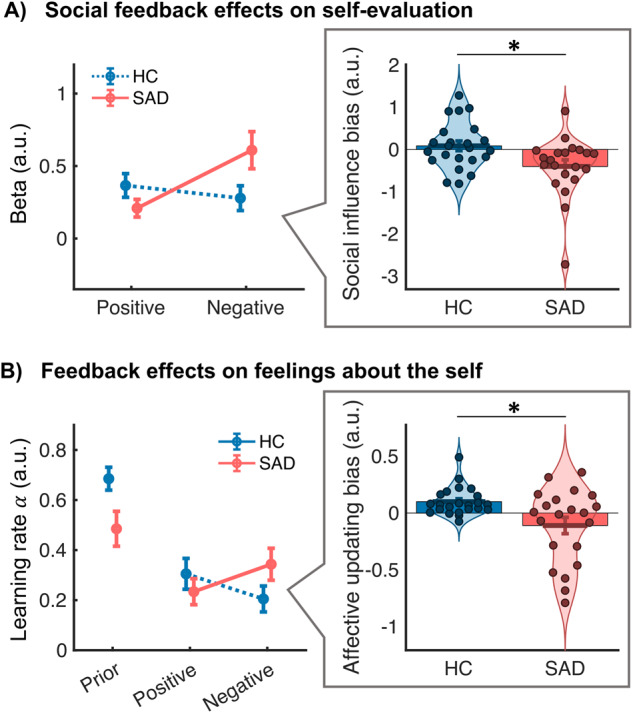
Behavioral results. **A** Beta weights (reflecting degree of learning from social feedback) for positive and negative feedback mismatch (*ΔEval*) in HC and SAD. Differences between positive and negative learning (‘social influence bias’) are shown in violin plots on the right (each dot reflects the value for one participant). Participants in the SAD group had significantly more negatively biased learning than those in the HC group. **B** Model-based prior value and learning rates for affective updating in HC and SAD. SAD showed significantly lower prior self-esteem than controls. Whereas HC showed a bias towards more positive affective updating, SAD participants had a more negative affective updating bias. Relative to HCs, their state self-esteem was more updated towards negative versus positive social feedback. Bars show group means and vertical lines indicate SEM.

Here, we build on these previous findings by further assessing the extent to which SAD is characterized by altered social learning of self-related information—specifically, the propensity to update more strongly from negative vs. positive social feedback—and by evaluating the brain mechanisms underlying these effects. We recruited individuals with SAD and matched control participants (total *N* = 44). All participants mentally prepared and gave a speech to be evaluated by judges. Then, across 52 trials, they rated their performance, received social feedback about their performance, and rated how they felt about themselves (Fig. 1A, B). After a break, they rated their performance a second time. This allowed us to evaluate how positive and negative social feedback: (1) caused changes in self-evaluation of performance (Fig. 1C) and (2) led to changes in state self-esteem, which we term ‘affective updating’ (see two example participants in Fig. 1D). We predicted that, compared to HC, SAD participants’ self-evaluations and their state self-esteem would be influenced more by negative than positive social feedback. We further predicted that these behavioral effects would be paralleled and mediated by activity in anterior insula, vlPFC, and anterior midcingulate cortex (aMCC)—often activated during social (and non-social) conflict and error detection [29]—and by negatively biased activation of areas related to social influence, updating, and cognitive control, including regions within the FPN, especially the dorsolateral prefrontal cortex (dlPFC) [26].

## Methods

### Participants

Forty-four adult participants took part in the present study (see Supplementary Information for detailed recruitment procedures). For all participants, we used the detailed Anxiety and Related Disorders Interview Schedule for DSM-5 [30] to assess SAD, and the briefer Mini International Neuropsychiatric Interview for DSM-5 [31] to assess additional diagnoses, administered by phone. For the SAD group (*N* = 21), we selected participants who fulfilled DSM-5 criteria for SAD (and not for any current mood, psychotic, or substance use disorder on the MINI, or any suicide attempts or major depressive episode in the past 5 years, to distinguish SAD effects from residual depression). For the healthy control group (HC, *N* = 23), we selected those who met the same clinical criteria except that in addition, they could not meet criteria for SAD or subthreshold SAD or any other current anxiety disorder. Demographic and clinical information is presented in Table S1. In brief, groups were matched for age, sex, self-reported race, education, and other demographic factors. In line with their difference in clinical status, they differed in expected directions on self-report questionnaires regarding anxiety, depressive, and ruminative symptoms (Table S1). Three additional participants were excluded from all analysis because of technical problems that led to substantial delays and made the manipulation implausible (*n* = 1), high number of missed trials due to falling asleep in the scanner (*n* = 1), and major movement artefacts (*n* = 1). All participants performed the speech preparation and speech in the fMRI scanner. The majority (*n* = 32; 16 SAD, 16 HC) also performed the feedback task in the scanner, while twelve performed it outside the scanner in a quiet testing room immediately after exiting the scanner (due to scanning constraints). The target sample size of 21 participants per group was determined based on a previously observed large effect size (partial η^2^ > 0.15, Cohen’s *d* = 0.8–0.9) [12], 80% power, and a two-sided significance threshold of *p* < 0.05. All participants provided informed consent and were paid for their time. The study protocol was approved by the institutional review board of the Department of Psychology and Neuroscience at the University of Colorado and in compliance with all relevant ethical regulations.

### Materials and procedures

#### Overview

Participants were told that their task would be to prepare and give a speech about their ideal job and why they were ideally suited for it. Participants were informed that two judges in the control room (who introduced themselves via the scanner’s interphone) would evaluate their speech and give them feedback after the speech. To increase believability, participants were briefly introduced to one of the judges in a lab coat in the scanner room before entering the scanner. Participants were given 4 min to mentally prepare the speech while undergoing fMRI scanning (data not analyzed here), followed by delivery of the 3-min speech in the scanner (during which no brain images were acquired). Experimenters were blind to diagnostic group during data acquisition.

#### Feedback task

Each of the 52 trials of the feedback task began with an evaluation cue—a short phrase describing a positive or negative aspect of their speech performance (see Fig. 1 and Table S2). Participants then used a visual analogue scale (VAS, coded as 0–1, anchored “completely disagree” and “completely agree”) to evaluate themselves regarding the content of this phrase (‘self-evaluation’). Then the judges’ feedback was presented, displayed as a second cursor line in a different color on the same scale and screen as the participants’ own evaluation. The ‘judges’ feedback’ was selected randomly from a distribution centered around the participant’s own self-evaluation. Approximately half of the trials had a positive and half of the trials had a negative feedback mismatch (*ΔEval*, the difference between the judges' feedback and participants’ self-evaluation). At the end of each trial, participants rated how they felt about themselves (‘state self-esteem’) on a VAS from 0–1.

Participants performed a second self-evaluation (T2) outside the scanner, approximately 20 min after the initial feedback task. T2 followed the same structure as the T1 feedback task, but contained only the evaluation cues and self-evaluative ratings (no judges’ feedback or self-esteem ratings), thus allowing us to test how the feedback received at T1 affected self-evaluation at T2. At the end of the experiment, participants were thoroughly debriefed, using a written debriefing questionnaire (see Figure S3) and a funneled verbal debriefing by the experimenter.

### Behavioral analysis

#### Feedback effects on self-evaluation

A multi-level general linear model (GLM) was used to assess the effect of positive and negative feedback mismatch (*ΔEval*) on changes in self-perception. Self-evaluation at T2 (*SelfEval(T2)*) was modeled as the sum of self-evaluation at T1 (*SelfEval(T1)*) and the product of the difference between the judges’ feedback and self-evaluation at T1 (*ΔEval*), weighted by individual beta weights (*β*, separately modeled for positive and negative feedback mismatch *ΔEval*):

$$\begin{array}{c}SelfEval(T2)=SelfEval(T1)+{\beta }_{pos}\varDelta Eval\begin{array}{cc}\quad for\; \varDelta Eval \,>\, 0\end{array}\\ SelfEval(T2)=SelfEval(T1)+{\beta }_{neg}\varDelta Eval\begin{array}{cc}\quad for \; \varDelta Eval \, <\, 0\end{array}\end{array}$$

For each participant, this approach yielded first-level (beta) estimates separately for positive and negative feedback mismatch values (related to prediction errors in standard reward learning tasks), reflecting how more positive versus negative feedback at Time 1 (relative to their own self-evaluation) influenced subsequent self-evaluations at Time 2.

#### Affective updating model

To characterize how state self-esteem was dynamically updated as a function of the judges’ feedback, we fitted an adapted reinforcement learning model [32], as in recent work [12]. Computational models [33–35] propose a concise description of self-related learning and potential biases, which can be formally tested in other studies. Comparisons between different models can also help to elucidate the underlying mechanisms of social anxiety disorder; further, models may provide a useful target for testing the effects of interventions and for the development of new treatments. In brief, state self-esteem in each trial *t* (*Feelings*_*Self*_*(t)*) is modeled as a function of self-esteem in the previous trial (*Feelings*_*Self*_*(t-1)*) plus the difference term (affective prediction error, *APE*) between self-esteem in the previous trial and the valence of the judges’ feedback in the current trial (*V*_*Feedback*_*(t)*), multiplied by a learning rate, *α*. The learning rate *α* reflects how strongly the judges’ feedback influenced current state self-esteem. Since we were interested in testing the differences in positive versus negative affective updating (i.e., how much self-esteem were driven by positive versus by negative feedback), we estimated separate learning rates for positive and negative *APE*s [12].

$$\begin{array}{c}Feelin{g}_{Self}(t)=Feelin{g}_{Self}(t-1)+\left\{\begin{array}{ccc}{\alpha }_{SelfPos}APE & for & APE \,>\, 0\\ {\alpha }_{SelfNeg}APE & for & APE\, <\, 0\end{array}\right.\\ where\quad APE={V}_{Feedback}(t)-Feelin{g}_{Self}(t-1)\end{array}$$

Model fitting was performed using the fmincon function of the Matlab Optimization toolbox by minimizing the sum of square errors between modeled and data time course. Free parameters (initial value of *Feeling*_*Self*_, *α*_*SelfPos*_ and *α*_*SelfNeg*_) were constrained to be between 0 and 1. Bayesian Information Criterion was used to assess the fit of a model with one versus with two (valence-specific) learning rates.

#### Statistical analyses

The data analysis plan was not preregistered but behavioral analysis were performed exactly as described previously [12]. All behavioral analyses were performed using MATLAB 2018b and custom code (https://github.com/canlab). Statistical comparisons used Student’s paired t-tests or Welch’s two-sample t-tests for unequal variances for group comparisons with a significance threshold of *p* < 0.05 (two-sided) unless otherwise specified.

### fMRI analysis

#### fMRI acquisition and preprocessing

Functional brain images were acquired using a Siemens TrioTim 3 T scanner (*n* = 16) and a Siemens Prisma 3 T scanner (*n* = 16, following a scanner update at the University of Colorado Boulder scanning facility). The proportion of patients and controls was identical before and after the update. Individual differences in signal largely outweighed differences in scanners, evidenced by the finding that adding scanner as a 2^nd^-level covariate did not meaningfully alter the results. A T2* weighted EPI GRAPPA sequence (TR = 1.3, TE = 25 ms, flip angle=50°, FOV = 220 mm) covered the brain in 26 interleaved transversal slices (3.4 mm isotropic voxels). SPM8 was used for preprocessing for functional images, using a standard pipeline of motion correction, slice-time correction, spatial normalization to MNI space, and spatial smoothing of images using an 8 mm FWHM Gaussian kernel. For spatial normalization, T1 structural MPRAGE images (1 mm isomorphic voxels) were first co-registered to the mean functional image and then normalized to the SPM template using unified segmentation. Preprocessed functional images were resampled to a voxel size of 3 × 3 × 3 mm.

#### General linear model

To characterize the brain responses to self-evaluative cues and feedback, we computed a general linear model (GLM) with regressors for: (1) the onset of the evaluative cue, (2) the self-evaluation rating, (3) the feedback of the judges, and (4) the self-esteem rating. A parametric modulator for *ΔEval* was added to the feedback regressor to model the size and direction of the mismatch between the participants’ self-evaluation and the judges’ feedback. Further, six movement regressors and their derivatives (per run), along with separate regressors corresponding to transient outliers (‘spikes’), were added as regressors of no interest. Robust regression was used to relate individual contrast maps from the GLM to individual differences in learning biases and to assess differences in brain responses to feedback and feedback mismatch between SAD and HC. To assess the activation of frontoparietal areas, we computed the similarity (Pearson correlation) of individual contrast images with a canonical mask of the frontoparietal network [36]. Similarity correlation coefficients were Fisher-z-transformed for statistical analyses.

#### Multilevel mediation analysis

To characterize the brain systems that mediated the effect of *ΔEval* on changes in self-evaluation, we performed a multi-level brain mediation analysis (https://github.com/canlab/MediationToolbox [37]). We first computed a single-trial GLM for each participant, containing separate regressors for each feedback trial (in addition to regressors for the other events across trials, as described above). The resulting beta-estimates for each feedback trial were then used in the multi-level brain mediation analysis. Brain mediation analysis formally tests three different effects to describe a potentially mechanistic neurobiological pathway from experimental manipulation (e.g., *ΔEval* at T1), via brain activity, to behavioral outcomes (e.g., changes in self-evaluation at T2). First, Path *a* tested the effect of the experimental manipulation (positive or negatively signed feedback mismatch, *ΔEval*) on brain activity, similar to the parametric modulator for *ΔEval* in the standard GLM. Second, Path *b* tested for brain activity related to the behavioral outcome (adjustments in T2 self-evaluation), when controlling for Path *a* effects. Third, Path *ab* (the mediation path) tested for brain activity that significantly mediated the effects of the feedback on changes in self-evaluation.

## Results

### Behavior

Participants’ self-evaluation at T2 was strongly influenced by the feedback they received at T1, as reflected in a significant main effect of feedback mismatch at T1 (*ΔEval*) on self-evaluation at T2 (*t(*42) = 13.71, *p* < 0.001, Cohen’s *d* = 2.12). Thus, overall, participants rated their performance as more positive at T2 when they had previously received more positive feedback, and more negatively, when they had previously received more negative feedback. The overall social influence effect (across positive and negative valence) was not significantly different between SAD and HC (*p* = 0.13). However, in line with our prediction and replicating our previous behavioral findings [12], the strength of the social influence effect was modulated by an interaction between group and valence (e.g., group difference in positive versus negative social influence effect, *t(*37.7) = 2.52, *p* = 0.016, Cohen’s *d* = 0.77, see Fig. 2A, B) such that SAD participants’ self-evaluation was influenced more by negative than by positive feedback mismatch (*t(*20) = −2.61, *p* = 0.017, Cohen’s *d* = 0.58), whereas HC showed a nonsignificant pattern of being influenced more by positive than negative feedback. Planned comparisons by feedback valence further confirmed that the SAD group was influenced more by negative feedback than HC (*t(*35.4) = 2.15, *p* = 0.039, Cohen’s *d* = 0.65), and a trend for the HC group to be more influenced by positive feedback than the SAD group (*t(*39.8) = 1.54, *p* = 0.13, Cohen’s *d* = 0.46).

A parallel pattern of results was observed for the model-based analysis of affective updating—how participants’ state self-esteem dynamically updated as a function of the feedback they received (see Fig. 2B and Figure S4 for model fit analyses). The initial value of state self-esteem was more negative for SAD than HC participants (*t*(35.0) = 2.40 *p* = 0.022, Cohen’s *d* = 0.73). Further, in line with our hypothesis, there was a significant interaction between group and valence of affective learning rates (group difference in difference between positive and negative updating, *t*(25.0) = 2.75, *p* = 0.011, Cohen’s *d* = 0.84) resulting from a significant positive updating bias in the HC group (i.e., a significant difference between *α*_*SelfPos*_ versus *α*_*SelfNeg*_, *t*(22) = 3.91, *p* < 0.001, Cohen’s *d* = 0.82), which was absent or even slightly (but not significantly) negative in the SAD group (*t*(20) = −1.52, *p* = 0.143, Cohen’s *d* = 0.33). For illustration, the time course of actual self-esteem ratings and of modeled self-esteem of two example subjects—one HC with a strong positive updating bias and one SAD with a strong negative updating bias—are shown in Fig. 1D.

Finally, individual differences in the affective updating bias (difference between positive versus negative affective learning rate) correlated positively with the bias in social influence on self-evaluation (difference between positive and negative social influence effect) (*r* = 0.54, *p* = 0.00014, see Figure S5). This relationship was significant even when controlling for group (partial correlation, *r* = 0.46, *p* = 0.0017). Thus, SAD participants with the most negative affective learning also showed the most negative bias for social influences on self-evaluation. This suggests that the two measures may reflect two interrelated aspects of more general individual differences in updating one’s self-concept. Both biases also correlated with questionnaires measuring anxiety and depression (see Figure S6 for details).

### fMRI results

#### Brain mediation analysis (across groups)

**Fig. 3:**
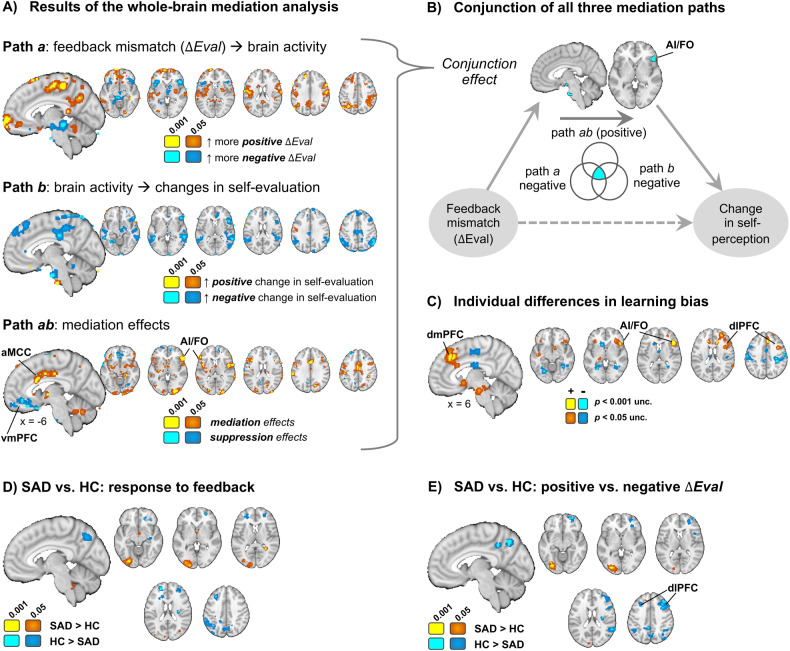
Whole brain imaging results. **A** Results of the mediation analysis, testing which brain areas mediate the effect of feedback mismatch (*ΔEval*) on changes in self-evaluation. Path *a* reflects activity associated with positive versus negative feedback mismatch. Path *b* reflects brain activity associated with changes in self-evaluation, controlling for path *a* effects. Path *ab* shows brain activity that formally mediates the effects of social influence on changes in self-evaluation. Note that yellow-orange colors reflect mediation (mirroring the direction of the direct effect), whereas blue colors reflect suppression effects, that can be interpreted as activity that ‘works against’ or is protective of social influence effects on self-evaluation. **B** Conjunction of all three mediation paths revealed a cluster in the right frontal operculum/vlPFC and brain stem. **C** Individual differences in learning bias (positive-negative) correlated positively with activity in bilateral AI/FO/vlPFC, dmPFC, dlPFC, striatum, and brainstem, and negatively with somatosensory and visual areas. **D** Differences between SAD and HC participants in overall response to the presentation of the judges’ feedback included precuneus, frontoparietal (SAD < HC) and occipital areas (SAD > HC). **E** Differences between SAD and HC participants in responses to signed (positive > negative) feedback mismatch (*ΔEval*) were found in frontoparietal areas (especially dlPFC and intraparietal sulcus), precuneus (SAD < HC), and occipital areas (SAD > HC).

To characterize the brain networks that mediate the effect of feedback mismatch on changes in self-evaluation across the two groups, we performed a multi-level brain mediation analysis with feedback mismatch (*ΔEval*) as the initial variable, fMRI activity in response to the feedback as the mediator [38, 39], and changes in self-evaluation as the outcome (see Fig. 3).

Path *a* effects—effects of more positive versus more negative *ΔEval*—were found in multiple areas previously associated with the processing of rewarding versus negative outcomes (see Fig. 3A). Specifically, more positive *ΔEval* was associated with activation in vmPFC, ventral striatum, precuneus, lateral prefrontal, and lateral parietal areas, whereas more negative *ΔEval* was associated with activation in bilateral anterior insula/frontal operculum (extending into the ventrolateral prefrontal cortex/vlPFC), thalamus, brainstem areas (periaqueductal gray) and other areas of lateral prefrontal cortex.

Path *b* effects—brain activation related to adjustments in self-perception (from T1 to T2) when controlling for Path *a* effects—were found mainly in lateral prefrontal and lateral parietal areas, anterior insula/frontal operculum, dorsomedial prefrontal cortex (dmPFC), rostral anterior cingulate cortex (rACC), and lateral temporal areas. These areas, except for a few small clusters in prefrontal and motor areas, all had negative weights, indicating they were related to adjustments towards more *negative* self-evaluation at T2.

Finally, Path *ab* (mediation) effects were found in dorsal ACC, anterior insula/frontal operculum (AI/FO), lateral prefrontal and parietal areas, as well as in several small clusters in the brainstem and the basal ganglia. In addition, suppression effects (negative Path *ab*, implying that the indirect effect via brain activity opposes the direct effects of feedback on self-perception updating) were found in vmPFC, suggesting that activity in this area may ‘work against’ or protect against social feedback effects on self-evaluation (Fig. 3A).

To identify brain areas that showed effects for all three mediation paths (Fig. 3B), we performed a conjunction analysis [40]. This revealed clusters in the right AI and adjacent FO and vlPFC, in the dorsolateral prefrontal cortex, and in the brainstem.

#### Correlation with individual learning bias (across groups)

Across both groups, individual differences in positive versus negative learning bias were positively correlated with positive versus negative feedback in AI/FO/vlPFC (overlapping with the mediation effects described above), dmPFC/aMCC, and dlPFC (yellow in Fig. 3C). Greater responses to negative (vs. positive) feedback in those areas was related to stronger negative (vs. positive) learning bias. Several other brain areas, especially the caudate and sensorimotor cortex, showed the opposite effect (see clusters illustrated in blue, Fig. 3C): More activity to negative (or less to positive) feedback was related to stronger positive learning bias.

#### Group differences

Using robust regression, we tested voxel-wise differences between HC and SAD groups in their responses to feedback and to valence of the feedback mismatch (Fig. 3D, E). At liberal thresholds, SAD compared to HC participants showed reduced responses to the presentation of the judges’ feedback (versus implicit baseline) in dmPFC, dlPFC, intraparietal sulcus, precuneus, and several smaller clusters, many of them in frontoparietal areas (Fig. 3D). A similar pattern of results was also observed for the group difference in the contrast for feedback mismatch (parametric modulator; Fig. 3E). Thus, those with SAD showed hypo-activation in precuneus and frontoparietal areas that also correlated with negative evaluative biases. At liberal thresholds, SAD compared to HC participants showed less lateral prefrontal and parietal activity for positive feedback relative to negative feedback mismatch. The amygdala showed a group difference in the same direction, suggesting it might be tuned more to negative (and less to positive feedback) in SAD compared to controls.

#### Responses of the frontoparietal network

**Fig. 4:**
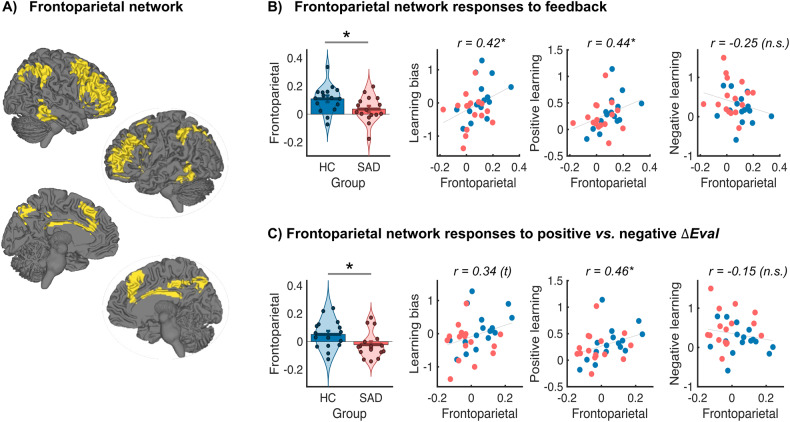
Activation of the frontoparietal network to feedback and feedback valence. **A** Display (in yellow) of a canonical frontoparietal network (FPN) mask [36]. **B** Activation of the frontoparietal network in response to feedback overall is significantly greater in HC than in SAD and correlates with positive learning bias. **C** HC compared to SAD show greater modulation of the FPN by positive versus negative valence of feedback mismatch (*ΔEval*), and the FPN modulation by valence correlates positively with the degree of positive (but not negative) learning. The dots in the violin plots show individual participants’ data, bars show group means, and vertical lines indicate SEM.

Given the role of frontoparietal areas in instruction and learning effects [26, 27], and impaired cognitive control and frontoparietal processes in anxiety and psychopathology more broadly [21, 41–43], we tested whether HC and SAD displayed differential responses in the frontoparietal network (Fig. 4A, parcellation by Yeo et al [36].). This analysis revealed that HC compared to SAD participants showed increased responses of the frontoparietal network to feedback overall (group difference, *t*(29.9) = 2.23, *p* = 0.034, Cohen’s *d* = 0.79, Fig. 4B). The response of the frontoparietal network correlated positively with individual differences in learning bias (positive > negative learning, Spearman’s *r* = 0.42, *p* = 0.017) and in positive learning based on feedback mismatch (Spearman’s *r* = 0.44, *p* = 0.013, see Fig. 4B).

Further, and line with our prediction, frontoparietal network responses in HC responded more to positive versus negative feedback mismatch (*ΔEval*), whereas SAD participants showed a slightly negative effect of positive versus negative *ΔEval* on frontoparietal responses (Fig. 4C, group difference: *t*(29.9) = 2.11, *p* = 0.043, Cohen’s *d* = 0.75). The relative activation of the FPN by positive versus negative feedback mismatch correlated with individual differences in learning bias (positive-negative learning, Spearman’s *r* = 0.35, *p* = 0.051) and in positive learning based on feedback mismatch (Spearman’s *r* = 0.46, *p* = 0.008, see Fig. 4C).

## Discussion

This study aimed to advance our understanding of the brain mechanisms underlying the altered processing of positive compared to negative self-related social feedback in participants with SAD compared to matched non-anxious controls. Negatively biased learning from self-relevant feedback appears to at least partially explain why those with SAD view themselves in a persistently negative way. The results contributed several insights to the growing literature of learning biases in SAD and other internalizing disorders. Behaviorally, we replicated the finding of negatively biased learning of self-related information demonstrated previously by our group and others [12–14, 44]. We then characterized the brain mechanisms underlying these differences. First, analyses of within-person updating across trials showed that the AI/FO/vlPFC mediates the influence of negative compared to positive social feedback on changes in self-evaluation. This effect also correlates with individual differences in negative updating bias across all participants but was not substantially different between SAD and HC. Second, we found that the ventromedial prefrontal cortex (vmPFC) suppressed social influence effects on changes in self-evaluation. Third, in HC, the FPN responded more to positive compared to negative social feedback, and in SAD, this frontoparietal positivity bias was absent if not reversed. This pattern of frontoparietal activation mirrored the differential social influence effects in those groups and correlated with individual differences in learning biases, and especially learning from positive social information. Together, these findings support a new neurobiological model of social learning of self-concept, and how social influences on the self are altered in SAD (and possibly related internalizing disorders), as discussed more below.

Our findings build on previous work examining the brain processes associated with changes in self-perception and self-esteem in healthy, non-anxious participants, who often have highly positive and sometimes inflated views of the self and their prospects [45–48]. For instance, Sharot and colleagues [47] showed that people learn more from positive than from negative information, and that individual differences in optimism bias were correlated with reduced tracking of negative estimation errors in right ventrolateral prefrontal cortex (vlPFC), located close to the frontal operculum and anterior insula cluster observed in the present results. In contrast to these positivity biases in updating beliefs, patients with major depression do not show a positivity bias and learn more from negative information than non-depressed controls [49, 50], which was paralleled by stronger responses to negative feedback in vlPFC/FO. Thus, this area, which is also involved in processing of social rejection [51, 52] and in emotion regulation more broadly [25, 38], appears to play an important role in responding to negative feedback and adjusting one’s self-image.

Similarly, Korn et al [45]. revealed a learning bias among healthy participants for updating self-perception of one’s personality traits more towards desirable than undesirable social feedback. Their findings further showed that activity in medial prefrontal cortex (mPFC) was associated with individual differences in this positive bias [45]. VmPFC activity has also been associated with social effects on valuation in other studies [53], including for influence by close others such as parents or peers [54], which is in slight contrast with the present finding of suppression of social influence effects in vmPFC. However, several other studies have suggested a role of the vmPFC/mOFC in positivity biases or resilience of self-related processing among healthy adults. For instance, mOFC activity is suppressed during social evaluative threat [37, 39], and this effect mediates activation of physiological threat responses [39] and stress-related impairment in cognitive performance [55]. VmPFC has also been shown to correlate with self-protective and flattering self-views, especially in response to social-evaluative threat [56, 57] and to mediate self-protective behavior following social feedback [58]. Our finding that vmPFC shows suppression effects for social influences on self-evaluation is consistent with this idea and with a more general role of this area in implicit emotion regulation [59], consolidation of social information [60], and self-esteem [61]. It also fits with recent computational findings suggesting that activation of positive self-schemas can buffer against negative learning about the self [14] and with evidence that the vmPFC tracks self-related value, whereas more dorsal regions of ACC track other-related value in social settings [62].

A key finding of our study is that the FPN responds differentially to feedback and especially to the valence of feedback mismatch in SAD compared to HC participants. Previous accounts have proposed that SAD is characterized by a decreased functioning of the frontoparietal and the default mode network [63], consistent with the idea that reduced emotion regulation is a core feature of this disorder. However, results have been mixed, with some studies reporting reduced FPN activity in SAD (e.g., during emotion regulation [64], while others reporting increased FPN activity [23]. One explanation for mixed results might be that in SAD, distinct areas of the prefrontal cortex are altered differently, or that the FPN shows altered activity only for SAD-relevant content or contexts [63]. In the present study, we found reduced activity in SAD that correlated with reduced learning from positive and enhanced learning from negative feedback. These findings converge with previous evidence that FPN activity mediates social information effects on pain [27]. A methodological advantage of our study is that we used an existing and established mask of the FPN and compared feedback- and valence-related activity across the entire network, yielding one value per participant, which can be easily compared across groups. Together, these findings further support a role of the FPN in social learning effects on behavior and experience [26, 27], and they show that this role is modulated by both the affective valence of the social information (relative to one’s own self-judgement) and by mental health conditions such as SAD. Our findings align with the recent proposal that the FPN plays a meta-cognitive and top-down regulatory role of self-related content [28].

**Fig. 5:**
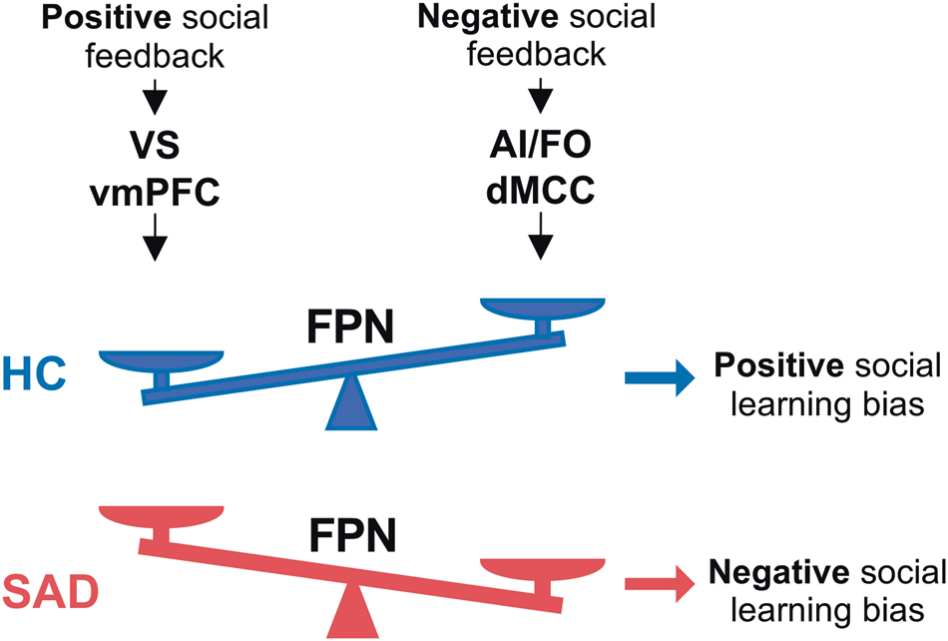
Schematic model of altered social influence effects in SAD. Positive versus negative social feedback is processed by VS/vmPFC and AI/FO/aMCC respectively, to a similar extent in both SAD and HC. However, FPN responses are positively biased, leading to more positive social learning in HC. This bias is absent or reversed in SAD, leading to an absence of positivity bias or even negative social learning about the self in this condition.

Integrating findings from the previous literature with the present results, we propose a new model of how social feedback alters self-perception and how this process might be altered in SAD (see Fig. 5). Insula and anterior midcingulate cortex (aMCC) detect negative social feedback prediction error, whereas areas related to positive reward prediction errors such as VS respond to more positive social feedback prediction error. Individual differences in the strength of positive and negative responses in these areas contribute to individual differences in affective biases. Frontoparietal areas may allocate attention to these prediction errors and update self-related representations based on social information, with a positivity bias for self-related information in controls, but not in socially anxious participants. DMN areas such as the vmPFC may reflect more intrinsic self-related processing and conceptualizations of self-in-context [65] that could buffer against external social feedback effects.

As a limitation, we note that our sample was relatively small and predominantly white, and thus our findings should be replicated in larger and more diverse samples. Future studies could also test this experimental paradigm in other anxiety disorders and investigate whether the present findings are specific to SAD or generalizable across different internalizing disorders when using task content that is relevant to a given disorder. Further, our speech and feedback paradigm likely was a robust stressor, especially for socially anxious individuals. Acute threat generally reduces reward [66] and increases punishment sensitivity [67]. In addition, stress can increase the observed differences between anxious and non-anxious people [67]. Thus, it remains an open question whether the acute stress evocation may have contributed to or enhanced group differences in self-related learning biases.

Future research should also test whether learning biases are stronger for evaluative statements that individuals are most concerned about and for negative compared to positive *statements* (e.g., ‘appeared nervous’ versus ‘appeared calm’), since previous work has shown that memory biases in SAD may differ between positive and negative items [68] and avoidance of negative impression might be more salient in SAD than the motivation to make a good impression.

In conclusion, this study advances the understanding of biased social learning for self-referential information in SAD by investigating the brain mechanisms underlying these effects. Our findings identify that anterior insula and frontoparietal systems (including dlPFC, lateral parietal areas, and parts of precuneus) mediate the effects of social feedback on self-perception, whereas vmPFC may buffer social feedback effects on self-perception. Future studies could evaluate the effect of therapeutic interventions on self-related learning, for example by testing whether interventions such as psychoeducation, cognitive-behavioral therapy, medication, or self-compassion interventions [69] reduce negative behavioral and frontoparietal biases. Future studies could also investigate whether the present effects generalize to other internalizing disorders and whether they constitute a transdiagnostic factor across different psychiatric conditions. If so, assessing biased processing of information related to the self and/or social feedback may help to fine-tune individual diagnosis and treatment strategies.

### Supplementary information

Supplemental material

## Data Availability

Deidentified aggregate data for this study is available upon request to the corresponding author. Code for analysis is available on canlab.github.org.
