# Correction: Brain mediators of biased social learning of self-perception in social anxiety disorder

**DOI:** 10.1038/s41398-023-02617-w

**Published:** 2023-11-17

**Authors:** Leonie Koban, Jessica R. Andrews-Hanna, Lindsay Ives, Tor D. Wager, Joanna J. Arch

**Affiliations:** 1 Lyon Neuroscience Research Center (CRNL), CNRS, INSERM, Université Claude Bernard Lyon 1, Bron, France; 2 Department of Psychology, University of Arizona, Tucson, AZ USA; 3 Department of Psychology and Neuroscience, University of Colorado, Boulder, CO USA; 4 Department of Cognitive and Brain Sciences, Dartmouth College, Hanover, NH USA

**Keywords:** Psychiatric disorders, Learning and memory

Correction to: *Translational Psychiatry* 10.1038/s41398-023-02587-z, published online 02 September 2023

The Funding section of this article was incomplete and should have included: Publication of this article was funded by the University of Colorado Boulder Libraries Open Access Fund and by internal CRNL funds to LK.

The original article has been corrected.

