# Supplementary material for: Brain mediators of biased social learning of self-perception in social anxiety disorder
Source: Transl Psychiatry. 2023 Sep 2;13:292. doi: 10.1038/s41398-023-02587-z (PMC10475036; doi:10.1038/s41398-023-02587-z)
Supplement: Supplementary file 1 — Supplemental material [file 41398_2023_2587_MOESM1_ESM.pdf]

Supplementary Information:

Brain mediators of biased social learning of self-perception in social anxiety disorder

Leonie Koban, PhD[1]*
Jessica R. Andrews-Hanna, PhD[2]
Lindsay Ives, PhD[3]
Tor D. Wager, PhD[4]
Joanna J. Arch, PhD[3]

[1] Lyon Neuroscience Research Center (CRNL), CNRS, INSERM, Université Claude Bernard Lyon 1, Bron, France
[2] Department of Psychology, University of Arizona, Tucson, AZ
[3] Department of Psychology and Neuroscience, University of Colorado, Boulder, CO
[4] Department of Cognitive and Brain Sciences, Dartmouth College, Hanover, NH

* correspondence to:
Dr. Leonie Koban, leonie.koban@cnrs.fr

Table S1. Participant characteristics.

	HC (N=23)	*SAD (N=21)*	*p-value*
**Gender** (M/F)	8/15	8/13	0.820
**Age** in years, M (±STD)	23.3 (±5.0)	23.9(±4.9)	0.691
**Education** in years, M (±STD)	15.6 (±2.7)	15.4 (±1.6)	0.839
**Race**			
African American	0	1	
Asian/Asian American	1	1	
Native American/Pacific Islander	1	0	
White	19	19	
Multiple	1	0	
Prefers not to respond	1	0	0.561
**Ethnicity**			
Hispanic or Latino	1	3	
Not Hispanic or Latino	21	18	
Prefer not to respond	1	0	0.342
**Employment**			
Unemployed	1	1	
Work part-time	1	3	
Work full-time	7	4	
Student part-time	3	1	
Student full-time	4	5	
Work full-time/student part-time	1	0	
Student full-time/work part-time	4	6	
Student full-time/work full-time	1	0	
Other	1	1	0.731
**Relationship**			
Single	9	6	
Divorced or separated	0	1	
In a relationship but not living together	7	8	
In a relationship and living together	3	3	
Married	4	3	0.787
**Community**			
Big city	3	3	
Suburb of a city	7	2	
Small city	12	14	
Small town	1	2	0.365
**Data**			
fMRI	16	16	
Behavior only	7	5	0.622
**BDI**	3.3 (±4.4)	9.7 (±9.6)	0.009*
**BNFE**	32.3 (±7.9)	46.5 (±9.8)	<0.001*
**BIS**	37.0 (±6.6)	40.2 (±6.2)	0.102
**CFQ**	36.3 (±8.4)	48.4 (±9.7)	<0.001*
**FFMQ**	137.5 (±12.4)	125.3 (±16.2)	0.008*
**IPI_MW**	33.4 (±7.0)	40.0(±6.9)	0.003*
**MASQ_AA**	11.7 (±2.3)	14.3 (±4.0)	0.014*
**NSPS**	41.3 (±13.2)	69.0 (±15.4)	<0.001*
**OASIS**	3.7 (±2.3)	9.9 (±3.0)	<0.001*
**PSWQ**	10.2 (±3.6)	17.2 (±4.6)	<0.001*
**RRQ**	33.1 (±7.7)	46.0 (±6.6)	<0.001*
**SCS**	96.0 (±16.2)	73.0 (±15.0)	<0.001*
**SHS**	22.7 (±3.4)	19.0 (±4.6)	0.005*
**SWLS**	28.3 (±3.8)	22.4 (±6.2)	0.001*
**LOT-R**	30.2 (±4.3)	25.8 (±6.2)	0.010*
**PRCS**	72.1 (±2.6)	67.1 (±3.2)	<0.001*

Note. HC = Healthy control participants, SAD = Participants with social anxiety disorder, BDI = Beck Depression Inventory[1], BNFE = Brief Fear of Negative Evaluation Scale[2], BIS = Barratt Impulsiveness Scale[3], CFQ = Cognitive Fusion Questionnaire[4], FFMQ = Five Facets Mindfulness Questionnaire[5], IPI_MW = Imaginal Process Inventory Mindwandering[6], MASQ_AA = Mood & Anxiety Symptom Questionnaire (Anxious Arousal Subscale)[7], NSPS = Negative Self-Portrayal Scale[8], OASIS = Overall

Anxiety Severity and Impairment Scale[9], PSWQ = Penn State Worry Questionnaire[10], RRQ = Rumination Reflection Questionnaire (Rumination subscale[11]), SCS = Self-compassion scale[12], SHS = Subjective Happiness Scale[13], SWLS = Satisfaction with Life Scale[14], LOT-R = Life Orientation Test Revised[15], PRCS = Personal Report of Confidence as a Speaker[16]. Welch's t-tests for unequal variances was used for group comparisons regarding age, years of education, and questionnaire scores. Chi-square tests were used for group comparisons on the remaining (categorical) variables.

Supplementary Methods

Participant recruitment. Participants were recruited using flyers on the CU Boulder campus and the larger Boulder community (e.g., local libraries, cafeterias, bus stops, grocery stores, gyms, …), via ads on Craig's List and social media (Facebook, twitter accounts), and ads on university listserves ('Buff's bulletin', Intermountain Neuroimaging Consortium). Interested individuals were invited to email the research team, and the research team responded by sending a link to a Pre-Screening questionnaire administered via RedCap. Those who passed the pre-screening were then interviewed by trained research assistants using the MINI for DSM-5 as well as the Social Anxiety Module of the ADIS-5 by phone to determine final study eligibility. To be included in the MRI study, participants had to be right-handed, between 18-40y old, have normal or corrected-to-normal vision, be able to read and write comfortably in English, own a personal smartphone with internet and texting capabilities (due to an experience-sampling procedure during eight days prior to the brain imaging part of the study, as reported elsewhere[17]), not have any neurological conditions or contra-indications for MRI scanning (e.g., pregnancy, metal implants, claustrophobia, pacemaker, cochlear implant, tattoos on head or neck, older tattoos with metal-containing inks, non-removable metal piercings, implanted infusion pump devices…).

General procedures. Prior to the task of interest in the current study, participants were scanned while completing a resting state task and a prospection task (findings to be reported elsewhere). They were then told that they had 4 minutes during which they should mentally prepare a 3-min speech on the topic: "Why would you be a good candidate for your ideal job?". Participants were further told that judges would listen to their speech and will later give them feedback on their speech. To increase believability of the speech task and feedback procedure, the participants were briefly introduced to one of the judges in a lab coat in the scanner room before entering the scanner. However, unbeknownst to the participants, the judges (confederates) did not actually evaluate the performance, and only one, female judge was in the control room (in addition to the experimenter and scanner operators). The other (male) judge was introduced based on a replayed voice recording which was piloted to be indistinguishable from a person present at the scan. One of the judges prompted the participant to start the speech. If the participant stopped talking for more than 20 seconds at any point, the judge reminded them to keep talking by saying "Please continue". After 3 minutes, one of the judges informed participants the speech task had ended. Following a brief questionnaire, participants then performed the two runs of the feedback task described in the *Methods* of the paper.

Feedback task. For each trial within the T1 feedback task, participants: 1) first rated themselves on their speech (self-evaluation), 2) then received the judges' feedback (while their own rating remained on screen, thus revealing the mismatch between their own rating and the judges' feedback), and 3) rated their current self-esteem. Approximately half of the trials had a positive and half of the trials had a negative feedback mismatch (*ΔEval*, the difference between the judges' feedback and participants' self-evaluation), with absolute values ranging from 0.17-0.44 (on the VAS scale from 0-1). On trials in which the judges' feedback would have been beyond the scale limits (smaller than 0 or greater than 1, which occurred when the participant rated themselves particularly low or high), the judges' feedback was replaced with a random value between the limit and the participants' self-evaluation.

Table S2. Evaluative statements used in the social feedback task. During the initial presentation of the statement and during the self-evaluative rating phase, the statements were preceded by “I” (e.g., “I was organized”). During the presentation of the judges’ feedback, the statements were preceded by “She” or “He” (e.g., “She was organized” or “He was organized”). The presentation order of statements was randomized for each subject. Reverse-coded items are marked with (-).

**Evaluative statement**	
appeared calm	
appeared charming	
appeared confident	
appeared genuine	
appeared inexperienced	(-)
appeared nervous	(-)
appeared relaxed	
appeared tense	(-)
blushed	(-)
communicated effectively	
had a steady voice	
handled the topic well	
held the judges' attention	
presented well	
rambled	(-)
seemed friendly	
seemed knowledgeable	
seemed reasonable	
seemed stressed	(-)
seemed sweaty	(-)
seemed thoughtful	
seemed uncomfortable	(-)
seemed uneasy	(-)
sounded anxious	(-)
sounded enthusiastic	
sounded impressive	
sounded polished	
sounded professional	
sounded shaky	(-)
spoke clearly	
spoke well	
stayed on topic	
trailed off at times	(-)
used the time well	
was awkward	(-)
was boring	(-)
was charismatic	
was competent	
was convincing	
was easily understood	
was eloquent	
was energetic	
was engaging	
was flustered	(-)
was guarded	(-)
was interesting	
was likeable	
was organized	
was pleasant	
was repetitive	(-)
was too serious	(-)
was upbeat	

Figure S3

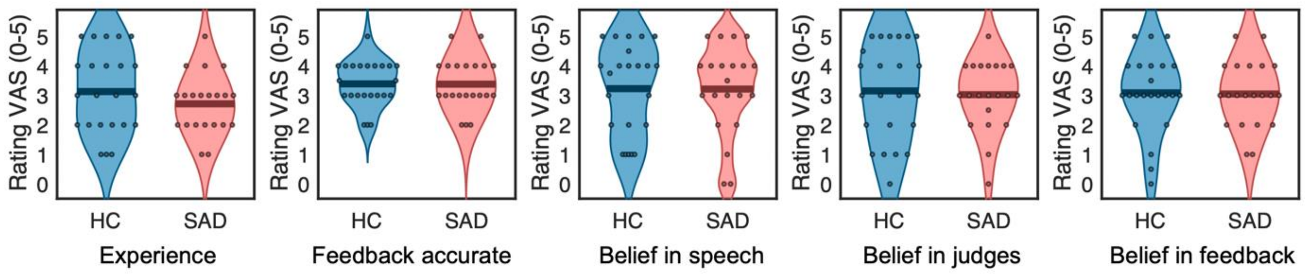

Figure S3. Results of the funneled debriefing questionnaire. From left to right, HC (blue) and SAD (red) participants' ratings on a visual analog scale (VAS, higher values reflect more agreement with statement) in 1) experience in public speaking, 2) perception that the feedback accurately described their performance, 3) belief that they would actually have to give a speech, 4) belief that they would be judged, and 5) belief that the feedback was real. Participants had a substantial range of experience with giving oral presentations (mean rating and standard deviation on a VAS from 0-5, M(STD) = 2.93(1.23)). Most of them described the feedback as rather accurate in describing their performance, M(STD) = 3.39(0.81). Regarding the oral debriefing questions, participants showed substantial variation but overall believed that they had to give a speech, M(STD) = 3.22(1.49), that they would be judged, M(STD) = 3.09(1.41), and that the feedback given was real, M(STD) = 3.07(1.21). Notably, from the oral debriefing it appeared that even those participants who had doubts about the feedback said they were affected by it, in line with previous results demonstrating that even hypothetical social feedback affects mood and brain responses[18]. Most importantly, none of the debriefing items differed significantly between HC and SAD groups (all p's > 0.26) and most of them (except 'Experience with public speaking') had almost identical mean values. Thus, any differences in behavior or brain responses between groups could not be explained by measurable differences in how much HC and SAD groups believed in the authenticity of the judges and the feedback.

Figure S4

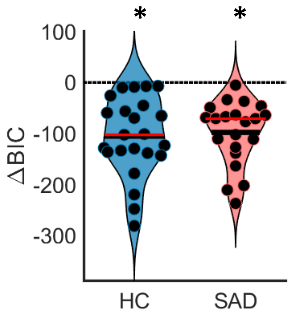

Figure S4. Comparison of affective updating models. Difference in model fits (as measured with the Bayesian Information Criterion/BIC) between the affective updating model with two learning rates (for positive and for negative updating separately) and a simpler model with one learning rate. The model with two learning rates had a lower BIC (indicating better model fit) for almost all participants in both healthy (HC) and socially anxious (SAD) participants. Dots indicate values for individual participants, black lines mean, and red lines median values per group.

Figure S5

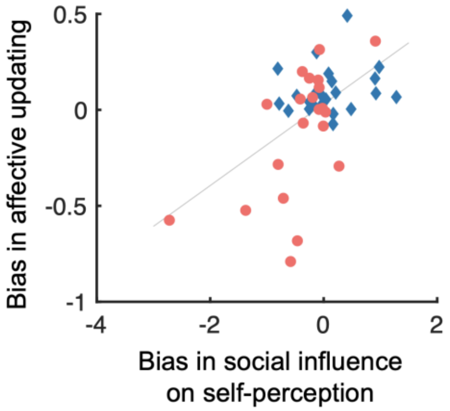

Figure S5. Correlation between positive-negative bias in social influence effects on self-perception with positive-negative bias in affective updating. HC are shown as blue diamonds, SAD participants as red circles.

Figure S6

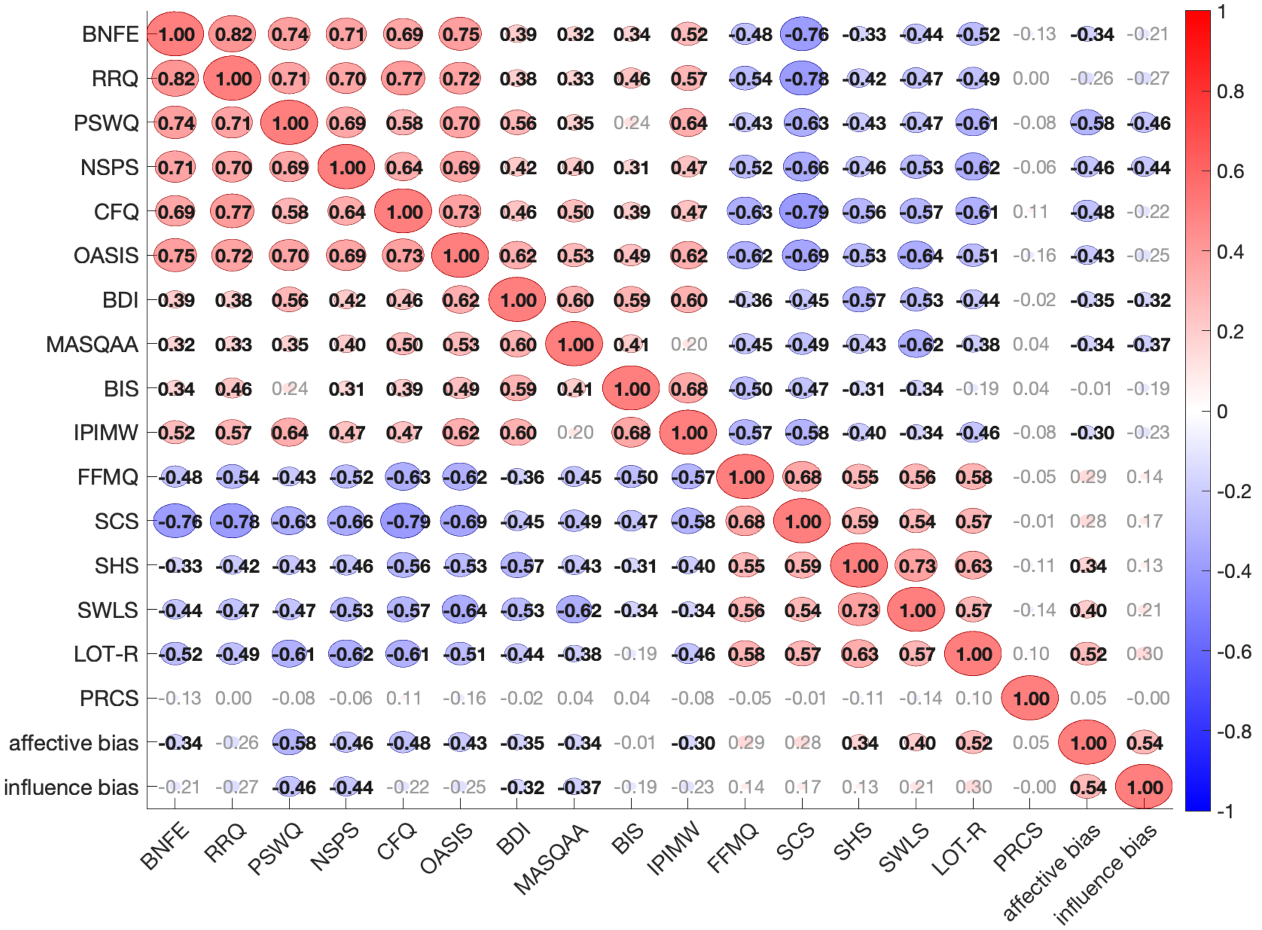

Figure S6. Correlations between questionnaire scores and learning biases (across groups). Bivariate Pearson correlation coefficients are displayed in a correlation matrix (questionnaires ordered based on a cluster analysis). Negative correlations are indicated by blue, positive correlations by red ellipsoids, with size of the ellipsoid reflecting the strength of the correlation. Significant correlations (p<0.05, uncorrected for multiple comparisons) are indicated by bold font. As expected, questionnaires measuring anxiety, depression (BDI, BFNE, BIS, CFQ, MASQAA, NSPS, OASIS, PSWQ, RRQ) and mindwandering (IPIMW) correlated positively with each other and negatively with questionnaires measuring self-compassion, resilience and optimism (SCS, SHS, SWLS, LOT-R). Affective updating bias correlated negatively with anxiety, mindwandering, and depression questionnaires, and positively with happiness, life satisfaction, and optimism. Bias in social influences on self-evaluation correlated negatively with anxiety and depression symptoms. To exclude the possibility that affective updating bias and social influence bias were driven more strongly by depression than anxiety scores, we added both NSPS and BDI in a GLM. This analysis showed that the effect of NSPS remained significant for both the affective updating bias ($t(41)=2.6$, $p=0.015$) and the social influence bias ($t(41)=2.45$, $p=0.019$), whereas the BDI score did not have significant effects on either bias (both p's > 0.2). BDI = Beck Depression Inventory[1], BNFE = Brief Fear of Negative Evaluation Scale[2], BIS = Barratt Impulsiveness Scale[3], CFQ = Cognitive Fusion Questionnaire[4], FFMQ = Five Facets Mindfulness Questionnaire[5], IPI_MW = Imaginal Process Inventory Mindwandering[6], MASQ_AA = Mood & Anxiety Symptom Questionnaire (Anxious Arousal Subscale)[7], NSPS = Negative Self-Portrayal Scale[8], OASIS = Overall Anxiety Severity and Impairment Scale[9], PSWQ = Penn State Worry Questionnaire[10], RRQ = Rumination Reflection Questionnaire (Rumination subscale[11]), SCS = Self-compassion scale[12], SHS = Subjective Happiness Scale[13], SWLS = Satisfaction with Life Scale[14], LOT-R = Life Orientation Test Revised[15], PRCS = Personal Report of Confidence as a Speaker[16], affective bias = positive – negative affective updating learning rate, influence bias = positive – negative social influence effect on self-evaluation.

Supplementary References

1. Beck AT, Steer RA, Carbin MG. Psychometric properties of the Beck Depression Inventory: Twenty-five years of evaluation. *Clin Psychol Rev* 1988; **8**(1)**:** 77-100.
2. Carleton RN, McCreary DR, Norton PJ, Asmundson GJG. Brief fear of negative evaluation scale-revised. *Depress Anxiety* 2006; **23**(5)**:** 297-303.
3. Patton JH, Stanford MS, Barratt ES. Factor structure of the Barratt impulsiveness scale. *J Clin Psychol* 1995; **51**(6)**:** 768-774.
4. Gillanders DT, Bolderston H, Bond FW, Dempster M, Flaxman PE, Campbell L *et al.* The development and initial validation of the cognitive fusion questionnaire. *Behav Ther* 2014; **45**(1)**:** 83-101.
5. Baer RA, Smith GT, Lykins E, Button D, Krietemeyer J, Sauer S *et al.* Construct validity of the five facet mindfulness questionnaire in meditating and nonmeditating samples. *Assessment* 2008; **15**(3)**:** 329-342.
6. Giambra LM. A factor analysis of the items of the imaginal processes inventory. *J Clin Psychol* 1980; **36**(2)**:** 383-409.
7. Watson D, Clark LA. Mood and Anxiety Symptom Questionnaire. *Journal of Abnormal PsychologyJournal of Behavior Therapy and Experimental Psychiatry* 1991.
8. Moscovitch DA, Huyder V. The negative self-portrayal scale: development, validation, and application to social anxiety. *Behavior Therapy* 2011; **42**(2)**:** 183-196.
9. Norman SB, Cissell SH, Means-Christensen AJ, Stein MB. Development and validation of an Overall Anxiety Severity And Impairment Scale (OASIS). *Depress Anxiety* 2006; **23**(4)**:** 245-249.
10. Meyer TJ, Miller ML, Metzger RL, Borkovec TD. Development and validation of the Penn State Worry Questionnaire. *Behav Res Ther* 1990; **28**(6)**:** 487-495.
11. Trapnell PD, Campbell JD. Private self-consciousness and the five-factor model of personality: distinguishing rumination from reflection. *Journal of personality and social psychology* 1999; **76**(2)**:** 284.
12. Neff KD. The development and validation of a scale to measure self-compassion. *Self and identity* 2003; **2**(3)**:** 223-250.
13. Lyubomirsky S, Lepper HS. A Measure of Subjective Happiness: Preliminary Reliability and Construct Validation. *Soc Indic Res* 1999; **46**(2)**:** 137-155.
14. Diener E, Emmons RA, Larsen RJ, Griffin S. The Satisfaction With Life Scale. *J Pers Assess* 1985; **49**(1)**:** 71-75.
15. Scheier MF, Carver CS, Bridges MW. Distinguishing optimism from neuroticism (and trait anxiety, self-mastery, and self-esteem): a reevaluation of the Life Orientation Test. *J Pers Soc Psychol* 1994; **67**(6)**:** 1063-1078.
16. Hook JN, Smith CA, Valentiner DP. A short-form of the Personal Report of Confidence as a Speaker. *Pers Individ Dif* 2008; **44**(6)**:** 1306-1313.
17. Arch JJ, Wilcox RR, Ives LT, Sroloff A, Andrews-Hanna JR. Off-task thinking among adults with and without social anxiety disorder: an ecological momentary assessment study. *Cogn Emot* 2021; **35**(2)**:** 269-281.
18. Hsu DT, Sanford BJ, Meyers KK, Love TM, Hazlett KE, Wang H *et al.* Response of the μ-opioid system to social rejection and acceptance. *Mol Psychiatry* 2013; **18**(11)**:** 1211-1217.
